# Inferring Temporal Information from a Snapshot of a Dynamic Network

**DOI:** 10.1038/s41598-019-38912-0

**Published:** 2019-02-28

**Authors:** Jithin K. Sreedharan, Abram Magner, Ananth Grama, Wojciech Szpankowski

**Affiliations:** 1 Center for Science of Information, Department of Computer Science, Purdue University, West Lafayette, IN, USA; 2 Department of Electrical Engineering and Computer Science, University of Michigan, Ann Arbor, MI, USA

## Abstract

The problem of reverse-engineering the evolution of a dynamic network, known broadly as network archaeology, is one of profound importance in diverse application domains. In analysis of infection spread, it reveals the spatial and temporal processes underlying infection. In analysis of biomolecular interaction networks (e.g., protein interaction networks), it reveals early molecules that are known to be differentially implicated in diseases. In economic networks, it reveals flow of capital and associated actors. Beyond these recognized applications, it provides analytical substrates for novel studies – for instance, on the structural and functional evolution of the human brain connectome. In this paper, we model, formulate, and rigorously analyze the problem of inferring the arrival order of nodes in a dynamic network from a single snapshot. We derive limits on solutions to the problem, present methods that approach this limit, and demonstrate the methods on a range of applications, from inferring the evolution of the human brain connectome to conventional citation and social networks, where ground truth is known.

## Introduction

Complex systems are comprised of interacting entities; e.g., cellular processes are comprised of interacting genes, proteins, and other biomolecules; social systems, of individuals and organizations; and economic systems, of financial entities. These systems are modeled as networks, with nodes representing entities and edges representing their interactions. Typical systems continually evolve to optimize various criteria, including function (e.g., flow of information in social networks, evolution of brain connectomes to specialize function), structure (e.g., evolution of social network structures to minimize sociological stress while maximizing information flow), and survivability (e.g., redundant pathways in genic interactions as evidenced by synthetic lethality screens). Recent results have also demonstrated advantages of dynamic networks in achieving quicker controllability^1^. Effective analysis of dynamic networks provides strong insights into the structure, function, and processes driving system evolution.

The problem of inferring the evolution of a dynamic network is of considerable significance: in a network of financial transactions, the arrival order of nodes tracks the flow of capital. In mapping spread of infectious diseases, node arrival order allows one to identify early patients, yielding clues to genetic origin, evolution, and mechanisms of transmission. In networks of biochemical interactions (e.g., protein interaction networks^2^) one can identify early biomolecules that are known to be differentially implicated in diseases^3^. Recently, strategic seeding and spread of (mis)information in online social networks like Twitter and Facebook has been hypothesized to create strong biases in opinions, even skewing electoral outcomes. Identifying sources and mechanisms of information transfer enables us to quarantine sources in a timely manner and to control spread.

## Our Contributions

We model and formulate the problem of recovering the temporal order of nodes in general graph models. Focusing on preferential attachment graphs and on deriving fundamental limits on inference of temporal order, we show that there exists no estimator for recovering temporal arrival order with high probability, owing to inherent symmetries in networks. Motivated by this negative result, we relax the formulation to admit a *partial order* on nodes. In doing so, we allow the estimator to make fewer vertex pair order inferences, in exchange for higher precision (e.g., by grouping nodes and finding the order only between groups but not within groups). We refer to the fraction of all node pairs that is comparable by a partial order (in terms of the arrival order) as the partial order density. We cast the partial order inference problem as a rational linear integer program, which allows us to present detailed analytical results on the achievable limits in terms of the tradeoff between expected precision and partial order density. To solve the optimization problem, we need to count the number of linear extensions of the partial order, which is known to be #P-complete^4^. There exists a fully-polynomial-time approximation algorithm that approximates the optimal solution to arbitrarily small relative error. However, in view of its significant computational cost, we propose a Markov chain Monte Carlo technique that achieves faster convergence in practice. We introduce and analyze, both theoretically and empirically, efficient estimators: our first estimator is optimal in the sense that it yields *perfect* precision. It infers all vertex order relations that hold with probability one. However, we find such relations to be asymptotically small compared to the total number of correct pairs. This motivates our investigation of other algorithms (the Peeling and Peeling+ algorithms), which sacrifice some precision in order to achieve higher density.

Experimental evaluation, on both synthetic and real-world datasets (network data of citations (ArXiv), collaborations (DBLP), hyperlinks (Wikipedia) and social connections (Facebook and SMS)), demonstrates the robustness of our methods to variations from the preferential attachment model. We also present a novel application of our method to the analysis of the human brain connectome to identify regions of “early” and “late” development. Our results reveal novel insights into the structural and functional evolution of the brain.

## Prior Works

The problem of inferring the sequence of node arrivals from a given network snapshot is highly complex, both analytically and methodologically, and has been little studied in prior literature. The works of Navlakha and Kingsford^5^ and Young *et al*.^6^ are the ones closest to ours. Navlakha and Kingsford^5^ formulates the problem as a maximum *a posteriori* estimation problem and develops a greedy algorithm for different graph models. Such a study can be translated to our maximum likelihood approach and we prove later that this leads to very large number of equiprobable solutions in the case of preferential attachment graphs. Young *et al*.^6^ studies the phase transition of recoverability via numerical experiments in the case of a non-linear preferential attachment graph in the Bayesian framework, with respect to the non-linear exponent of degree. Such a phase transition can be formally justified with the theoretical results in the Supplementary Material of this paper. Some prior results focus on variants of the problem of finding the oldest node in a graph^7,8^. The results of Bubeck *et al*.^7^ are only applicable to trees, thus severely limiting their application scope. Our proposed methods target general graphs and seek node orders beyond identifying the oldest node. Frieze *et al*.^8^ study the problem of identifying the oldest node in preferential attachment graphs using a local exploratory process with the assumption that the time index of a node can be retrieved once it is sampled. A related problem of detecting information sources in epidemic networks has been studied by Shah *et al*.^9^ and Zhu *et al*.^10^ for the Susceptible-Infected model. We first formulated the node arrival order inference problem and presented some preliminary results in ref.^11^.

## Results

**Figure 1:**
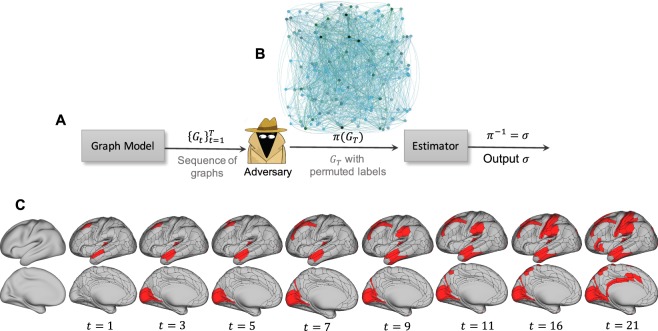
(**A**) Block diagram of our formulation. (**B**) A network of human brain formed from Human Connectome Project (HCP) data. This network is shown as an example of *π*(*G*_*T*_). Since the estimator should not dependent on the permutation, applying an unknown adversary permutation on node labels is equivalent to making the graph unlabeled. (**C**) Human brain evolution deduced by our method: Starting from network data in (**B**), we apply our techniques and make an inference on how brain regions evolve in the left hemisphere of a human brain. The time instant *t* in the figure represents an instant of change in the evolution of brain regions. The data and code are available at^24^. See Fig. 5 and the Methods for more details.

Let *G* be a graph of *n* vertices corresponding to a snapshot of a growing network, generated by a dynamic graph model. Without loss of generality, we count time in units of vertex additions. Since *G* has *n* vertices, we say that this is the snapshot at time *n*. We label vertices in their arrival order, [*n*] = {1, …, *n*}, where node *j* is the *j*th node to arrive. Note that these vertex labels are not known to us. Instead, the vertices are randomly relabeled according to a permutation *π* drawn uniformly at random from the set of permutations on *n* letters *S*_*n*_, and we are given the graph *H*: = *π*(*G*). Our goal is to infer the arrival order of vertices in graph *G* from observed graph *H*, i.e., to find the inverse permutation *π*^−1^, which reveals the true arrival order. See Fig. 1 for an illustration of our approach and an application on inferring the evolutionary order of prominent human brain regions. We provide further analyses of this result later in the paper.

We consider a general scenario in which we do not restrict our analysis to inference of a total order. Rather, we consider an estimator *ϕ* that outputs *partial orders* on the set of vertices (see Fig. 1A for an example). For a partial order *σ*, a relation *u* <_*σ*_*v* defined on vertices *u* and *v* means that vertex *u*’s label is less than that of vertex *v* in the partial order *σ*. We say that an ordered pair of vertices (*u*, *v*) in *π*(*G*) satisfying *u* < _*σ*_*v* forms a *correct pair* if *π*^−1^(*u*) < *π*^−1^(*v*); i.e., vertex *u* precedes vertex *v* in the true arrival order. Given a partial order *σ*, we can always algorithmically find a total order consistent with *σ* (i.e., a linear extension of *σ*).

We formally define measures for quantifying the performance of any estimator. For a partial order *σ*, let *K*(*σ*) denote the number of pairs {*u*, *v*} that are comparable under *σ*: i.e., *K*(*σ*) = |{(*u*, *v*) : *u* < _*σ*_*v*}|, where $|K(\sigma )|\le (\genfrac{}{}{0ex}{}{n}{2})$.

### Density

This is the number of comparable pairs in *σ* normalized by the total number of pairs, that is, $\delta (\sigma )=\frac{K(\sigma )}{(\genfrac{}{}{0ex}{}{n}{2})}.$ The density of a partial order estimator *ϕ* is thus $\delta (\varphi )={{\rm{\min }}}_{H\in {{\mathfrak{G}}}_{n}}[\delta (\varphi (H))]$, where ${{\mathfrak{G}}}_{n}$ is the set of all graphs of size *n*.

### Precision

This measures the expected fraction of *correct* pairs out of all pairs dictated by the partial order. That is,

$$\theta (\sigma )={\mathbb{E}}\,[\frac{1}{K(\sigma )}|\{u,v\in [n\mathrm{]\ :\ }u{ < }_{\sigma }\,v,{\pi }^{-1}(u) < {\pi }^{-1}(v)\}|].$$

For an estimator *ϕ*, we denote by *θ*(*ϕ*) the quantity ${\mathbb{E}}[\theta (\varphi (\pi (G)))]$.

### Recall

This measures the expected fraction of correct pairs (out of the total number of pairs) output by an algorithm inferring a partial order *σ*, that is,

$$\rho (\sigma )={\mathbb{E}}\,[\frac{1}{(\genfrac{}{}{0ex}{}{n}{2})}|\{u,v\in [n]:u{ < }_{\sigma }\,v,{\pi }^{-1}(u) < {\pi }^{-1}(v)\}|].$$

**Figure 2:**
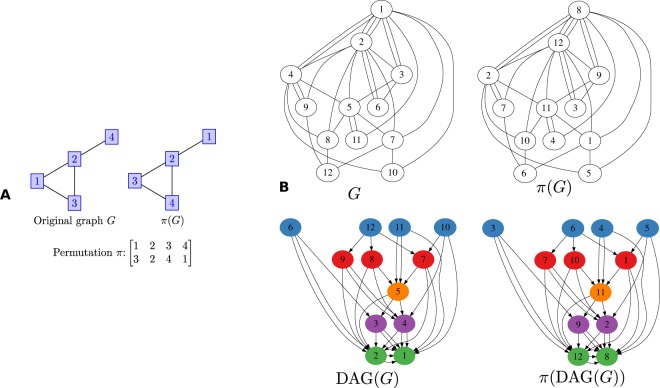
(**A**) An example scenario. The estimator sees only *π*(*G*) and must infer *π*^−1^. E.g., it may output the order $$\sigma =\{4\,\prec \,1\,\prec \,2\}$$. The relation $$4\,\prec \,1$$ is correct, since *π*^−1^(4) = 3 < *π*^−1^(1) = 4, but the relations $$4\,\prec \,2$$ and $$1\,\prec \,2$$ are incorrect, since *π*^−1^(4) = 3 > *π*^−1^(2) = 2 and *π*^−1^(1) = 4 > *π*^−1^(2) = 2. The density is $$\delta (\sigma )=3/(\tfrac{4}{2})=$$$$\mathrm{3/6}=\mathrm{1/2}$$, the precision is *θ*(*σ*) = 1/*K*(*σ*) = 1/3, and the recall is *ρ*(*σ*) = *θ*(*σ*) *δ* (*σ*) = 1/6. (**B**) The original graph (left) and the observed graph (right) for an instance of *π*: the same bin nodes in the DAGs have the same colors. Note that DAG(*G*) and *π*(DAG(*G*)) have exactly the same structure. The *π*(DAG(*G*)), generated by Peeling algorithm recovers all the probability one order information of *G*.

Figure 2A presents a sample graph, the permuted vertex labels, a candidate partial order, and measures of density, precision, and recall for the partial order.

We present our key results and illustrate them in the context of dynamic networks generated by Barabási-Albert preferential attachment model. Many dynamic networks arising in a variety of applications are hypothesized to follow this model of “rich-gets-richer” mechanism^12–18^. We denote a dynamic graph generated by the preferential attachment model as ${\mathscr{P}}{\mathscr{A}}(n,m)$^12^, where *n* is the number of nodes and *m* is the number of connections a new node makes to existing nodes when it is added to the network. At *t* = 1 a single vertex (labeled 1) is created with *m* self loops. To construct graph *G*_*t*_ at time 1 < *t* ≤ *n*, vertex *t* joins the network and makes *m* independent connections to the existing nodes in graph *G*_*t*−1_ with probability ${\rm{\Pr }}[t\,{\rm{connects}}\,{\rm{to}}\,k|{G}_{t-1}]=\frac{{{\rm{\deg }}}_{t-1}(k)}{2m(t-\mathrm{1)}},$ where deg_*t*−1_(*k*) is the degree of node *k* at time *t* − 1. Let DAG(*G*) be the directed acyclic version of *G* with the direction of edges marked in accordance with the graph evolution (leading from younger nodes to older nodes). For *π*(*G*), edge directions are captured in its directed version *π*(DAG(*G*)). Note that DAG(*G*) and *π*(DAG(*G*)) have the same structure. This is illustrated in Fig. 2B.

If we restrict the estimator to output a *total order*, i.e., *δ*(*σ*) = 1, we show in SM Section 3 that no algorithm can solve the problem with error probability asymptotically bounded away from 1. As a specific instance of a solution procedure, one may also frame the problem in terms of maximum likelihood estimation as follows:

$${{\mathscr{C}}}_{{\rm{ML}}}(H)=arg\,ma{x}_{\sigma \in {S}_{n}}{\rm{\Pr }}[{\pi }^{-1}=\sigma |\pi (G)=H\mathrm{]}.$$

We show that the set ${{\mathscr{C}}}_{{\rm{ML}}}$ yields a large number of equiprobable solutions, $|{{\mathscr{C}}}_{{\rm{ML}}}|={e}^{n\mathrm{log}n-O(n\mathrm{log}\mathrm{log}n)}$ with high probability, and therefore the maximum likelihood formulation is unsuitable. (*f*(*x*) = *O*(*g*(*x*)) indicates that there exist *δ* > 0 and *M* > 0 such that |*f*(*x*)| ≤ *M*|*g*(*x*)| for |*x* − *a*| < *δ*).

### Formulation and solution of the underlying optimization problem

In view of this negative result, we consider estimators outputting a *partial order* on nodes. Here, an estimator may make fewer vertex pair order inferences, in exchange for higher precision (e.g., by grouping nodes and inferring the order only across groups, but not within groups). We then seek an optimal estimator in the following sense: for an input parameter *ε* ∈ [0, 1], we seek an estimator *ϕ* with density *δ*(*ϕ*) ≥ *ε* and maximum possible precision *θ*(*ϕ*). This yields an optimal curve θ_*_(*ε*), that characterizes the tradeoff between precision and density. We derive computable bounds on this curve and present efficient heuristic estimators that approach the bounds.

Given a graph *H*, define the function *J*_*ε*_(*ϕ*) as the fraction of correctly inferred vertex orderings from among all allowable orderings by a given partial order. That is,

$${J}_{\varepsilon }(\varphi )=\frac{{\mathbb{E}}[|\{u,v\in [n]:u\, < \,{}_{\varphi (H)}v,{\pi }^{-1}(u) < {\pi }^{-1}(v)\}||\pi (G)=H]}{K(\varphi (H))},$$

and the conditional expectation is with respect to the randomness in *π* and *G*. To exhibit an optimal estimator, it is sufficient to choose, for each *H*, a value for *ϕ*(*H*) (i.e., a partial order) that maximizes the expression *J*_*ε*_(*ϕ*) subject to the density constraint, $K(\varphi (H))\ge \varepsilon (\genfrac{}{}{0ex}{}{n}{2})$. We can then write the precision of estimator *ϕ* as:

$$\theta (\varphi )=\sum _{H}{\rm{\Pr }}[\pi (G)=H]{J}_{\varepsilon }(\varphi \mathrm{)}.$$

To construct an optimal estimator, for each ordered pair (*u*, *v*) of vertices of *H*, we associate a binary variable *x*_*u*,*v*_, where setting *x*_*u*,*v*_ = 1 indicates that *u* < *ϕ*_(*H*)_*v*. We can then rewrite *J*_*ε*_(*ϕ*) as:

1 $${J}_{\varepsilon }(\varphi )=\frac{{\sum }_{1\le u < v\le n}\,{p}_{u,v}(H){x}_{u,v}}{{\sum }_{1\le u\ne v\le n}\,{x}_{u,v}},$$

where ${p}_{u,v}(H)={\rm{\Pr }}[{\pi }^{-1}(u) < {\pi }^{-1}(v)|\pi (G)=H]$ is the probability that *u* arrived before *v* given the permuted graph *H*, with the following constraints coming from the partial order and from our constraint on a given minimum density:

1. Antisymmetry: *x*_*u*,*v*_ + *x*_*v*,*u*_ ≤ 1.
2. Transitivity: *x*_*u*,*w*_ ≥ *x*_*u*,*v*_ + *x*_*v*,*w*_ − 1 for all *u*, *v*, *w* ∈ [*n*].
3. Minimum density: $${\sum }_{1\le u\ne v\le n}{x}_{u,v}\ge \varepsilon (\genfrac{}{}{0ex}{}{n}{2}).$$
4. Domain restriction: *x*_*u*,*v*_ ∈ {0, 1} for all *u*, *v* ∈ [*n*].

**Figure 3:**
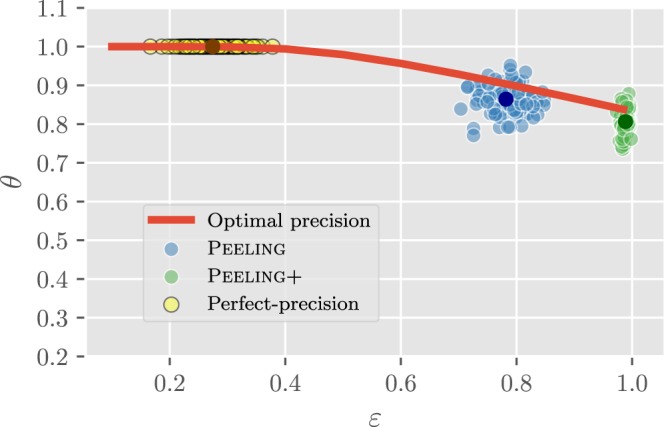
LP relaxation to the optimal precision curve θ_*_(*ε*) and estimators for $G\sim {\mathscr{P}}{\mathscr{A}}(n=50,m=3)$. The bold points indicate averaged value. The proposed three estimators serve different purposes. The perfect-precision estimator outputs pairs with full accuracy, but only a few. The Peeling+ gives a total order, but with less accuracy (which is much better than random guessing, and close to the optimal algorithm). The Peeling stands in the middle with better accuracy than Peeling+, and yet recovers a constant fraction of number of pairs.

We efficiently upper bound the optimal precision for any given density constraint *ε* as follows: on a randomly generated input graph *H* = *π*(*G*), we recover its edge directions (i.e., *π*(DAG(*G*))) and use them to approximate the coefficients *p*_*u*,*v*_(*H*) up to some relative error. The resulting rational linear integer program with approximated *p*_*u*,*v*_(*H*) can be converted into an equivalent linear integer program using a standard renormalization transformation, and we consider its natural linear programming relaxation with *x*_*u*,*v*_(*H*) ∈ [0, 1] for all *u*, *v*. This can be solved in polynomial time using standard algorithmic tools. We show the nature of this bound in Fig. 3.

To characterize the probability *p*_*u*,*v*_(*H*) and thus to solve the optimization, we prove that for all *u*, *v* ∈ [*n*] and graphs *H*

$${p}_{u,v}(H):={\rm{\Pr }}[{\pi }^{-1}(u) < {\pi }^{-1}(v)|\pi (G)=H]=\frac{|\{\sigma \,:\,{\sigma }^{-1}\in {\rm{\Gamma }}(H),{\sigma }^{-1}(u) < {\sigma }^{-1}(v)\}|}{|{\rm{\Gamma }}(H)|},$$

where the subset Γ(*H*) ⊂ *S*_*n*_ consists of permutations *σ* such that *σ*(*H*) has positive probability under the distribution ${\mathscr{P}}\,{\mathscr{A}}(n,m)$ (see SM Lemma 4.1). Thus the estimation of *p*_*u*,*v*_(*H*) can be reduced to counting linear extensions of the partial order given by *π*(DAG(*G*)), which is known to be #P-complete (ruling out an efficient exact algorithm). However, we propose a Markov chain Monte Carlo algorithm that achieves sufficiently fast convergence in practice.

### Exact recovery of edge directions

Given access to *H* = *π*(*G*), the following algorithm, which we call the Peeling technique, efficiently recovers *π*(DAG(*G*)) (thus the edge directions) for a graph *G* (see Fig. 1B). The algorithm starts by identifying the lowest-degree nodes (in our model, the nodes of degree exactly *m*), which are grouped into a bin. Then, it removes all of these nodes and their edges from the graph. The process proceeds recursively until there are no more nodes. To construct *π*(DAG(*G*)) during this process, we note that all of the edges of a given degree-*m* node in a given step of the Peeling process must be to older nodes; hence their orientations can be recovered. In the SM Section 6.1, we show that *π*(DAG(*H*)) captures all the probability-1 information about vertex orderings in *H* and Peeling exactly recovers *π*(DAG(*H*))

### Estimators

Due to the high polynomial time complexity involved in solving the optimal scheme (estimating the upper bound requires $O({n}^{5}{\mathrm{log}}^{3}n)$ calculations), we now provide efficient estimators whose performance is close to the optimal curve (see SM Section 6.2 for detailed analysis). In fact, the linear program itself does not yield an optimal scheme (one has to do a rounding step, which only yields an approximation) or an estimator, but only an upper bound on the optimal precision. Moreover, converting it to an optimal estimator is potentially computationally difficult, and thus efficient heuristics are needed.

1. *Maximum-density precision* 1 *estimator*: The estimator itself takes as input a graph *π*(*G*) and outputs the partial order as *π*(*DAG*(*G*)) (all connected node pairs with order as the direction of the connection) as recovered by the Peeling algorithm. This estimator gives the maximum density among all estimators that have precision one; however, as shown in Theorem 6.2 of SM, we only can recover *o*(*n*^2^) correct pairs.
2. Peeling *- A linear binning estimator via peeling*: When the term Peeling is used as an estimator, we mean the serial binning estimator from the bins (groups of nodes) given by the Peeling technique. In particular, the sequence of subsets of vertices removed during each step naturally gives a partial order: each such subset forms a bin, and bins that are removed earlier are considered to contain younger vertices (see Fig. 2B). The Peeling estimator, which returns the bins, outputs strictly more vertex pair order guesses than the optimal precision-one estimator. In particular Θ(*n*^2^) pairs, but some are not guessed correctly, and thus sacrifices some precision for increased density
3. Peeling+, *Peeling with deduction of same bin pairs*: This estimator runs on top of the Peeling estimator and attempts to order nodes within bins/groups. For each node, we find the averaged value of its neighbors’ bin numbers (levels), which we call the node’s *average neighbor level*. A high value of average neighbor level indicates youth of the node. For each pair of nodes inside each bin, we infer the order between them based on the the averaged neighbor level of the respective nodes.

Figure 3 compares these estimators with the optimal one based on the integer programming formulation above. These estimators are observed to have performance close to optimal, at different points on the optimal curve. *Furthermore, the time complexity of these estimators is dominated by the DAG construction, and is O*(*nlogn*).

### Experiments

In what follows, *σ*_perf,_*σ*_peel_, *σ*_peel+_ denote the partial orders produced by the Perfect-precision, Peeling, and Peeling+ estimators.

#### Robustness of the Peeling algorithm

Table 1 demonstrates robustness of our Peeling algorithm for various generalizations of the model: preferential attachment model with variable *m* (denoted by *M* and ~unif{*a*, *b*} denote discrete uniform distribution), uniform attachment model (denoted by ${\mathscr{U}}{\mathscr{A}}$), and the more general Cooper-Frieze model^19,20^. In our instance of the Cooper-Frieze model, the number of new edges *m* is drawn from ~unif{5,50}, the model allows either addition of a new node (with probability 0.75) or addition of edges between existing nodes, and the endpoints of new edges can be selected either preferentially (with probability 0.5) or uniformly among existing nodes. These results suggest that the proposed DAG-based methods can simultaneously achieve high precision and recall/density.

**Table 1:** A general comparison: *n* = 5000.

Technique	*θ* (*σ*_peel_)	*ρ* (*σ*_peel_)	*δ* (*σ*_peel_)
${\mathscr{P}}{\mathscr{A}}(n,m=25)$	0.958	0.936	0.977
${\mathscr{P}}{\mathscr{A}}(n,M)$, *M* ~ unif {5, 50}	0.691	0.683	0.988
${\mathscr{U}}{\mathscr{A}}(n,m=25)$	0.977	0.967	0.99
${\mathscr{U}}{\mathscr{A}}(n,M)$, *M* ~ unif {5, 50}	0.827	0.823	0.995
Cooper-Frieze (Web graph) model	0.828	0.822	0.993

#### Real-world networks

We now discuss the performance of our estimators on several real-world networks, presented in Table 2 We first consider the ArXiv network as a directed network, with nodes corresponding to publications and edges from each publication to those that it cites. We also analyze the Simple English Wikipedia network – a directed graph showing the hyperlinks between articles of the Simple English Wikipedia. The DBLP computer science bibliography data is then modeled as an undirected network; an edge between two authors represents a joint publication. Finally, we study an SMS network and an online social network of Facebook focused on the New Orleans region, USA, with an edge (*u*, *v*, *t*) representing that user *u* posted on user *v*’s wall at time *t*. Results are presented in Table 2. For all of the networks tested, the methods described here yield excellent precision and density.

**Table 2:** Results for real-world networks: A detailed description of the datasets are given in SM.

Dataset	# Nodes	# Edges	Genre	*θ* (*σ*_peel_)	*ρ* (*σ*_peel_)	*δ* (*σ*_peel_)	*ρ* (*σ*_peel+_)
ArXiv High Energy Physics	7.46 K	116 K	Citation	0.708	0.681	0.961	0.707
Simple English Wikipedia	100 K	1.62 M	Hyperlink	0.624	0.548	0.878	0.609
DBLP CS bibliography	1.13 M	5.02 M	Coauthorship	0.785	0.728	0.927	0.764
Facebook Wall post	43.9 K	271 K	Social	0.698	0.657	0.941	0.687
SMS network	30.2 K	447 K	Social	0.669	0.610	0.912	0.621

*θ* (*σ*_peel+_) ≈ *ρ* (*σ*_peel+_) and *δ* (*σ*_peel+_) ≈ 1. When the density of the recovered partial order by Peeling algorithm is low, the recall can be improved via Peeling+ with a slight loss in precision (see the Wikipedia result).

**Figure 4:**
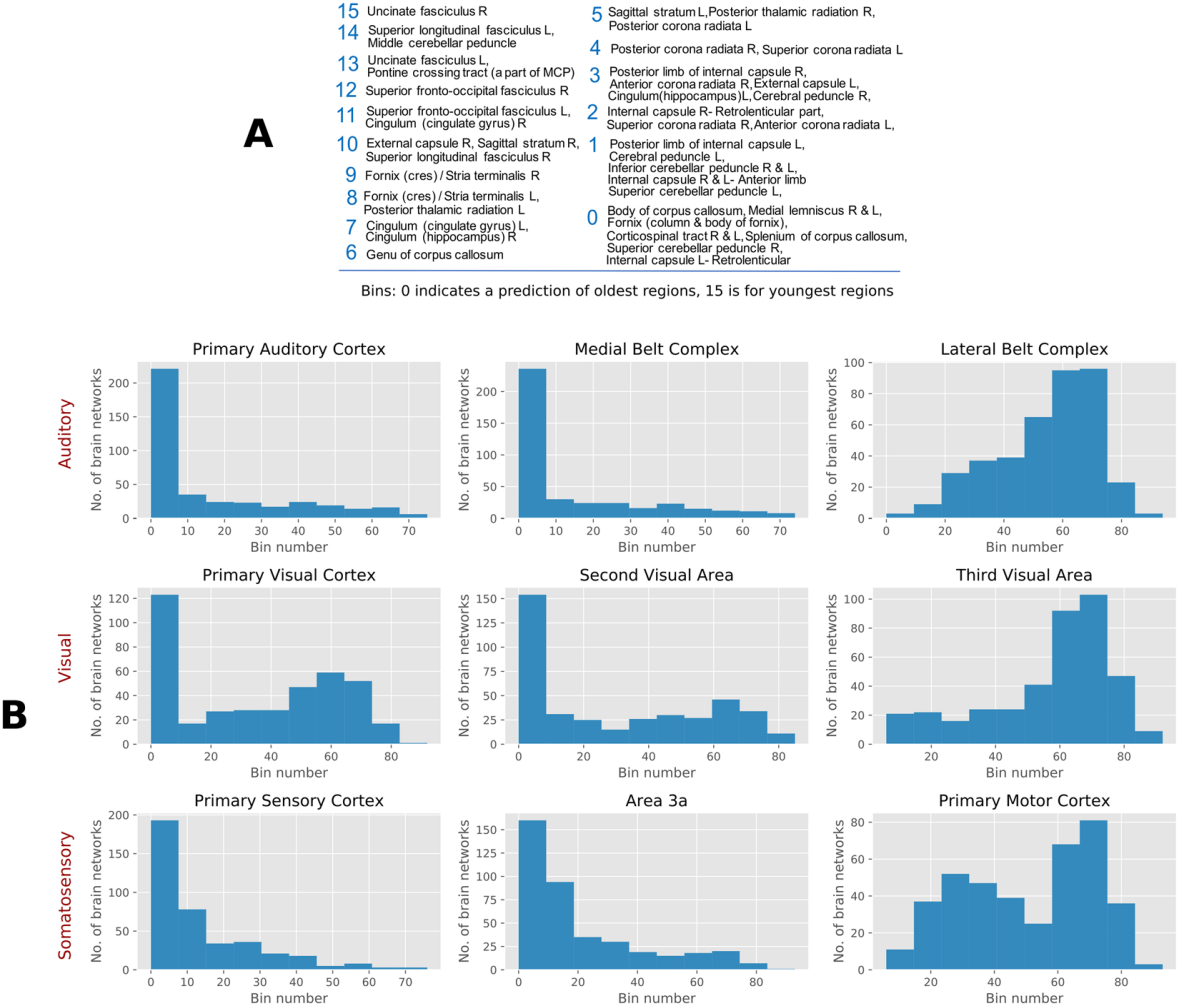
(**A**) Peeling bins of regions in Human brain network - Cambridge-Buckner. (**B**) Human Connectome brain data: histogram of selected regions in cortex.

**Figure 5:**
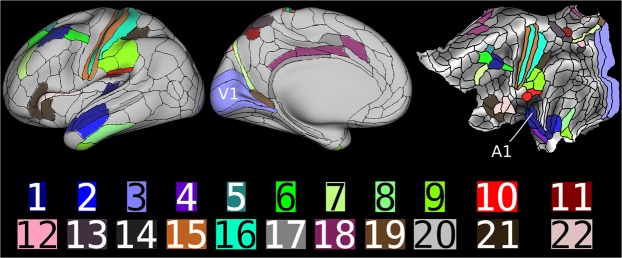
Illustration of the bins of prominent brain regions according to their arrival order in brain evolution: Inflated and flattened representation of left hemisphere of human brain. The network is formed from a correlation matrix of fMRI image of the data from Human Connectome Project (See Methods for details). The borders and regions are defined according to the multi-modal parcellation technique proposed in^22^, and the figure is generated with Human Connectome Project’s workbench tools. The node (brain region) ranking given by the Peeling algorithm are used to create batches of arrivals. The number indicated in the figure represents the first 22 bins, with bin 1 corresponding to the bin of oldest nodes. Note that the primary visual cortex (V1) and primary auditory cortex (A1) are classified into older bins. Code and data available at^24^.

Figures 4 and 5 presents results of our analysis on human brain networks. The purpose here is to recover the evolutionary order among the important regions inside the brain. We note that there is no available ground truth (in terms of the network) for such ranking. Therefore our ranking provides important insight for further application studies. However, our ranking is “supported” by prior application studies^13,21^, indicating that the brain network is well approximated by the preferential attachment mechanism and its variations. We study two independent sets of brain networks of resting state fMRI images. The first one is derived from the Cambridge-Buckner dataset with 56 labeled brain regions. The Peeling estimator provides a ranking of the brain regions; we analyze this ranking with respect to relatively sparse state of the art in our biological understanding of evolution of human brain. The corpus callosum, which joins the two hemispheres of the brain is believed to have developed in the earliest stages of brain evolution, and the uncinate fasciculus, the white matter tract to have evolved late in the human brain. These observations are consistent with the rankings returned by our Peeling estimator. Our rankings represent a first step towards determining the complete evolutionary trajectory of various regions of the brains.

The second network is extracted from the Human Connectome Project (HCP). We consider rankings of regions of 400 networks from 100 individuals (2 session and 2 scans per subject), with more detailed 300 labeled regions in cortex. We plot the histogram of arrival rank of prominent auditory, visual, and somatosensory regions. These histograms show a concentration of rankings, indicating that our rankings are consistent among 100 people in the regions considered. Moreover, we observe that most of the regions that serve as prime functionaries among auditory, visual, and somatosensory regions have consistently low arrival order in the 400 brain networks we analyzed. This is consistent with the widely accepted notion of early arrivals of these regions in the human brain evolution. Figure 5 illustrates the bins of brain regions of left hemisphere deduced with the Peeling estimator on a network generated from HCP data.

## Discussion

We focus here on node arrival inference from a single snapshot of a dynamic network. Our models, analyses framework, and methods are applicable to a broad class of dynamic network generation models. Our infeasibility results (details in the SM) of total order recovery are useful in understanding fundamental limitations owing to different types of symmetries in networks. The general optimization problem we pose, which includes solutions of total and partial orders, provides an overarching framework within which disparate algorithms can be evaluated. We use this framework to argue near-optimal solutions from our estimators.

In a broader perspective, network archeology is not limited to the recovery of node arrival order, it generalizes to inference of higher order structures like triads, motifs, and communities. Our work provides the foundation for a rich class of problems in the area, both from analytical and applications’ points of view. An alternate perspective of network archeology is in finding the course of (mis)information spreading. Recently, strategic information dissemination in online social networks like Twitter and Facebook has been alleged to create biases in opinions, even to the point of skewing electoral outcomes. Often, it is not one single source controlling the spread, rather, a group of nodes working in collusion. Our solutions provide powerful tools in identifying and quarantining these malicious nodes rapidly.

## Methods

### Code and data availability

We make our code available at https://github.com/jithin-k-sreedharan/times. The code supports random graph model (variants of preferential attachment model) and real-world networks. It also includes a script for generating brain networks from fMRI correlation data. The brain networks data is shared in the above link and the other networks used in this work are publicly available online. We solve the linear programming optimization using the Python interface of a commercial optimizer Gurobi, and the script is available in the same link.

### Constructing the brain networks

The data of Human Connectome project is the resting state fMRI data from Human Connectome project focusing on the cortex area. The Human Connectome project provides a clean and refined data, which gives consistent results in many published studies. We process and form brain networks out of 100 healthy young adults. First, the cortex brain data corresponding to 100 subjects (2 sessions per subject, and 2 scans per session) is parcellated into 180 regions per hemisphere using a procedure described by Glasser *et al*.^22^. Then the correlation matrices are formed from the time series of the parcellated data. Finally binary, undirected networks are constructed from the correlation matrices as follows: a spanning tree is created first from the complete network of the correlation matrix, and later *k*-nearest neighbors (higher correlation values) of each node are added into this network, where *k* is chosen as 10 in our case. Each network has 300 nodes, which are regions or clusters formed from group-Independent Component Analysis. The data is in correlation matrix format, with each element as the Gaussianized version of the Pearson correlation coefficient (Fisher Z transform with AR(1) process estimation). In order to form a binary adjacency matrix, we use a threshold just high enough to make the resulting graph connected. Such a graph is sparse.

### Estimating *p*_*u*,*v*_ using Markov chain Monte Carlo

We describe the procedure to estimate the integer programming coefficients *p*_*u*,*v*_ = *p*_*u*,*v*_(*H*) (${\rm{\Pr }}[{\pi }^{-1}(u) < {\pi }^{-1}(v)|\pi (G)=H]$). Solving the original optimization requires knowledge of *P* = [*p*_*u*,*v*_], which can be estimated via MCMC (Markov chain Monte Carlo) techniques. The order of convergence of one important MCMC technique (which we will call the Karzanov-Khachiyan algorithm and denote by K-K) for sampling uniformly from the set of linear extensions of a partial order, reported by^23^, is *O*(*n*^6^ log(*n*)log(1/*ε*)) transitions to achieve *ε*-bounded error between the distribution of a sampled linear extension and the uniform distribution. Estimation of certain functions of the set of linear extensions in general requires more transitions. For instance, Brightwell and Winkler^4^ proved that estimating the total number of linear extensions based on K-K chain requires $O({n}^{9}\,{\mathrm{log}}^{6}(n))$ transitions. From a practical, computational perspective, this time complexity is untenable. Thus, we propose a different random walk (RW)-based algorithm.

First, a linear extension graph is formed as *G*_LE_ = (*V*_LE_, *E*_LE_), where vertex set *V*_LE_ consists of linear extensions Γ(*H*) consistent with the partial order (DAG) given by the DAG. The extensions *λ* and *μ* are adjacent in the graph if and only if *λ* can be obtained from *μ* by an adjacent transposition. We describe a RW process below, which does not require the graph to be known beforehand, instead the graph will be explored locally as neighbors of the nodes sampled by the random walk.

**Figure 6:**
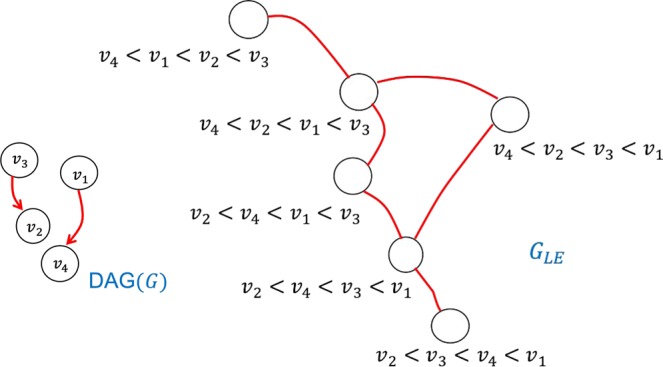
An example DAG(*G*) and its linear extension graph *G*_LE_.

For instance, let {*v*_1_, *v*_2_, *v*_3_, *v*_4_} be the nodes of the underlying PA graph. Let the partial order given by the DAG be *v*_2_ < *v*_3_ and *v*_4_ < *v*_1_. Then a node in the linear extension graph (which is a linear extension with the given partial order) is *v*_4_ < *v*_1_ < *v*_2_ < *v*_3_. Among the three possible adjacent transpositions of this total order, only one is a linear extension, which is *v*_4_ < *v*_2_ < *v*_1_ < *v*_3_. Thus the degree of this total order is 1. Figure 6 shows the graphs DAG(*G*) and *G*_LE_ for this example.

The algorithm is as follows:

1. We sample a node *λ* in *G*_LE_, which is a linear extension, using the Sequential algorithm (The Sequential algorithm works similar to the Peeling technique, but instead of Peeling away all the *m*-degree nodes at each step, it removes only a randomly selected node among the *m*-degree nodes present at any step and all other nodes stay for the next removal.). Let it be the initial node.
2. The neighbor set of *λ* can be obtained as follows. If any adjacent elements in *λ* form a perfect pair, they are not allowed to swap positions. All other adjacent pairs are allowed to transpose, and each neighbor of *λ* corresponds to a linear extension differed from *λ* with one transposed pair.
3. We run a simple random walk on graph *G*_LE_ with the random walk choosing the next node in the walk uniformly among the neighbors of the present node.
4. Such a RW has a stationary distribution $d(\lambda )/{\sum }_{\mu \in {\rm{\Gamma }}(H)}d(\mu )$, where *d*(*λ*) is the degree of linear extension *λ* in *G*_LE_. We form the following ratio form estimator for *P*_*u*,*v*_ without directly unbiasing such a non-uniform distribution, which is impossible without the knowledge of ${\sum }_{\mu \in {\rm{\Gamma }}(H)}d(\mu )$.
  $${\hat{p}}_{u,v}^{(k)}:=\frac{\sum _{t=1}^{k}{\bf{1}}\{{X}_{t}(u) < {X}_{t}(v)\}/d({X}_{t})}{\sum _{t=1}^{k}\mathrm{1/}d({X}_{t})}\,\mathop{\longrightarrow }\limits^{k\,\to \,\infty }\,\frac{|\mu \in {\rm{\Gamma }}(H):\mu (u) < \mu (v)|}{|{\rm{\Gamma }}(H)|}\,{\rm{a}}.{\rm{s}}.$$
  Here *X*_*t*_ indicate the *t*-th sample of the RW, which is a linear extension of the underlying DAG.
5. Stop the RW when a convergence criteria is met.

Note that, unlike in K-K method, we do not need to make Markov chain aperiodic as the intention is not to sample from the unique stationary distribution, but only to estimate an average function of the nodes. The K-K method first forms a Markov chain similar to the construction in the above algorithm, but makes it aperidoc by adding self transitions with probability 1 − *d*(*λ*)/(2*n* − 2). Given the discussion in SM Section 6.2.1, we expect *d*(*λ*) should be very small, and hence the Markov chain in the K-K method spends a large amount of time in self loops, thus making the mixing slower. Our method avoids artificial self loops, and achieves faster convergence in practice.

## Supplementary information

Supplementary Material
