# Supplementary material for: Inferring Temporal Information from a Snapshot of a Dynamic Network
Source: Sci Rep. 2019 Feb 28;9:3057. doi: 10.1038/s41598-019-38912-0 (PMC6395620; doi:10.1038/s41598-019-38912-0)
Supplement: Supplementary file 1 — Supplementary Material [file 41598_2019_38912_MOESM1_ESM.pdf]

Supplementary Material: Inferring Temporal Information from a Snapshot of a Dynamic Network

Jithin Sreedharan[†], Abram Magner[†], Ananth Grama, and Wojciech Szpankowski[*]

Center for Science of Information, Purdue University, USA
[*]To whom correspondence should be addressed
E-mail: szpan@purdue.edu
[†]Authors contributed equally.

February 3, 2019

Contents

1 Introduction **2**
1.1 Overview of prior work 2

2 Model and Definitions **2**

3 Infeasibility of Total Order Recovery **3**
3.1 Maximum likelihood estimation 6

4 Formulation and Solution of an Optimization Problem **7**
4.1 Reduction of rational integer program to linear program 7
4.2 Coefficients of optimal precision 8

5 Optimal Solution for Preferential Attachment Graph Model **8**
5.1 Recovering edge directions 8
5.2 Integer program coefficients via $\mathrm{Dir}(H)$ 9

6 Algorithms and Estimators **10**
6.1 Exact recovery of $\pi(\mathrm{DAG}(G))$ 10
6.2 Estimators and their properties 12
6.2.1 Maximum-density precision 1 estimator 12
6.2.2 Peeling: A linear binning estimator via peeling 15

7 Experiments **15**
7.1 Synthetic graphs 15
7.2 Real-world networks 16
7.2.1 Results 18

1 Introduction

Throughout the supplementary information, we will generally follow certain notational conventions (unless otherwise stated), which we describe here. An arbitrary random graph model on n vertices is denoted by $\mathcal{G}_n$, and a sample from that model by G. The random permutation generated by an adversary is given by π, and we use H to denote the observed graph $\pi(G)$ (a random variable) or one of its possible values. Fixed graphs will generally be denoted by capital letters.

1.1 Overview of prior work

Mahantesh et al. [15] empirically studied inference of arrival order via a binning method. In this context, we provide a rigorous formulation and analysis, a simpler solution without having to generate samples from an estimated random graph model. We also provide a precise characterization of the performance of our methods. To the best of our knowledge, our work presents the first feasible and rigorous approach to the problem of inferring partial orders in preferential attachment graphs. We first formulated the present problem and presented some preliminary results in [14, 13].

For preferential attachment graphs, Frieze et al.[6] study the problem of identifying the oldest node. However, their setting and goal are rather different: they assume that the arrival order of nodes is known, and their goal is to arrive at the oldest node by a process that starts at a different node and only uses local information to advance.

2 Model and Definitions

The given formulation is equivalent to the following intuitive scenario: we are given a graph G with unique vertex ids, and there exists a hidden bijection ψ from the set of ids to the integers $\{1, ..., n\}$ giving the order of arrival of the vertices, identified by their names. The task is to use the structure of G to recover ψ. Formally, then, the problem that we study is entirely specified by the following data: a distribution on labeled graphs (for the bulk of this paper, the preferential attachment distribution $\mathcal{PA}(n, m)$, but in general denoted by $\mathcal{G}_n$), the form that a solution takes, and a way of evaluating the performance of a given solution method.

To present succinctly our results we need three quantities associated with permutations of a graph model:

(a) *Set of feasible permutations of a graph H*: the subset $\Gamma(H) \subseteq S_n$, which consists of permutations σ, such that $\sigma(H)$ has positive probability under the distribution $\mathcal{PA}(n, m)$. An example of a permutation that is *not* feasible for a graph G generated by preferential attachment is $\pi = (1, n)$, which swaps the first and last vertices. This is because the degree of the vertex labeled n in the resulting graph $\pi(G)$ is $> m$, which happens with probability zero. We know that for this model $\mathbb{E}[\log |\Gamma(G)|] = n \log n - O(n \log \log n)$ [11].

(b) *Set of admissible graphs of H*: $\mathrm{Adm}(H) = \{\sigma(H) : \sigma \in \Gamma(H)\}$. These are graphs obtained by applying $\Gamma(H)$ to H.

(c) *Automorphism group*: The *automorphism group* of a graph G, denoted by $\mathrm{Aut}(G)$, is the set of permutations π of vertices of G that preserve edge relations (i.e., the number of edges between vertices u and v is equal to the number of edges between vertices $\pi(u)$ and $\pi(v)$ in G). Note that $|\mathrm{Adm}(H)| = |\Gamma(H)|/|\mathrm{Aut}(H)|$ [11].

3 Infeasibility of Total Order Recovery

Here we restrict the estimator to output a total order (i.e., a permutation) for which $\delta(\sigma) = 1$. An estimator function takes the form $\phi : \mathfrak{G}_n \to S_n$ and $\mathfrak{G}_n$ is the set of graphs on n vertices.

Let the minimax risk for a random graph model $\mathcal{G}_n$ be denoted by

$$R_*(\mathcal{G}_n, d) = \min_{\phi} \max_{\mathfrak{A}_n} \mathbb{E}[d(\phi(\pi(G)), \pi^{-1})],$$

where $\mathfrak{A}_n$ is the the set of all adversaries, and d is some distortion measure on permutations. We focus on two natural choices for d, namely, error probability $d_e(\sigma_1, \sigma_2) = I[\sigma_1 \neq \sigma_2]$ for exact recovery, and Kendall τ distance $\tau(\sigma_1, \sigma_2) = \sum_{1 \leq i < j \leq n} I[\sigma_2\sigma_1^{-1}(i) > \sigma_2\sigma_1^{-1}(j)]$ for approximate recovery.

The next result says that, at least for the two distortion measures described above, the worst-case adversary is the uniform distribution.

Theorem 3.1. *In the case of error probability and Kendall τ distortion measures, we have, for any adversary $\mathfrak{A}_n$, that the minimum risk over any estimator is greater than or equal to that for the adversary that chooses a permutation uniformly at random.*

Proof. Consider an arbitrary adversary that outputs a permutation π on input G. We will construct a randomized estimator as follows: we draw uniformly at random a $\sigma \sim S_n$ and compute $H' = \sigma(\pi(G))$. Note that $\sigma \circ \pi$ is uniformly distributed on S_n. The estimator will then apply an optimal estimator for the uniform adversary to H', yielding a permutation λ, which is meant to be an approximation of $(\sigma \circ \pi)^{-1} = \pi^{-1} \circ \sigma^{-1}$. Finally, the estimator outputs $\lambda\sigma$ as an approximation of π^{-1}.

To analyze the precision of this estimator, we note the following chain of equalities holds, by elementary properties of the Kendall τ distance:

$$\tau(\lambda\sigma, \pi^{-1}) = \tau(\lambda, \pi^{-1}\sigma^{-1}) = \tau(\lambda, (\sigma\pi)^{-1}). \tag{1}$$

Since $(\sigma\pi)^{-1}$ is uniform on S_n and λ was generated by an optimal estimator for the uniform adversary, the expectation of the final expression is exactly equal to the minimum possible risk for the uniform adversary.

Finally, standard results yield that there exists a deterministic estimator with at least as large risk, which concludes the proof. The derivation for the error probability distortion measure is similar, and we omit it. □

The following theorem gives a general upper bound on the recall.

Theorem 3.2. *Consider a random graph model $\mathcal{G}_n$ for which any two positive-probability isomorphic graphs are equiprobable. Then the minimax risk is*

$$\begin{aligned} R_*(\mathcal{G}_n, d_e) &\geq \frac{\mathbb{E}[\log|\mathrm{Aut}(G)|] + \mathbb{E}[\log|\mathrm{Adm}(G)|] - 1}{\log n!} \\ &= \frac{\mathbb{E}[\log|\Gamma(G)|] - 1}{\log n!}. \end{aligned}$$

Proof. We consider a particular adversary, one that chooses π uniformly at random from S_n. Applying Fano's inequality, we have that the probability of error is

$$\begin{aligned} \Pr[\phi(\pi(G)) \neq \pi^{-1}] &\geq \frac{H(\pi^{-1}|\phi(\pi(G))) - 1}{\log|S_n|} \\ &= \frac{H(\pi^{-1}|\phi(\pi(G))) - 1}{\log n!} \end{aligned}$$

Now, by the data processing inequality and chain rule,

$$\begin{aligned} H(\pi^{-1}|\phi(\pi(G))) &\geq H(\pi^{-1}|\pi(G)) \\ &= H(G|\pi(G)) + H(\pi^{-1}|G, \pi(G)). \end{aligned}$$

We note here that $H(\pi^{-1}|G, \pi(G)) = \mathbb{E}[\log |\mathrm{Aut}(G)|]$ as there are exactly $|\mathrm{Aut}(G)|$ automorphisms between G and $\pi(G)$, and $H(G|\pi(G)) = \mathbb{E}[\log |\mathrm{Adm}(G)|]$ since all isomorphic graphs with positive probability have the same probability. □

For the preferential attachment model, the following lemma proves the assumption in Theorem 3.2. Strictly speaking, we prove the lemma for the version of the model in which vertex degrees are updated with every connection choice. An approximate version holds under tweaks of the model (essentially because multiple edges are rare, and the results that rely on the lemma only require a very approximate version). In the proof and elsewhere throughout the supplementary information, we will use some common terms: a *DAG* is a directed, acyclic graph. The *structure* or *unlabeled* version of a graph or a DAG M (denoted by $S(M)$) is the isomorphism class of the object in question (i.e., the set of all graphs or DAGs that are equivalent to the given one, up to permutations of its labels).

Lemma 3.1. *Consider two isomorphic preferential attachment graphs* $G_1, G_2 = \sigma(G_1)$*,* $\sigma \in \Gamma(G_1)$*. If* $\Pr[G = G_1] > 0$*, then*

$$\Pr[G = G_1] = \Pr[G = G_2]. \tag{2}$$

Proof. First, we will show that for a positive-probability graph K, $\Pr[G = K]$ depends only on the unlabeled DAG of K (see Definition 5.1 for the definition of the labeled version – namely, an edge from u to v exists in the (labeled) DAG of K if and only if $u > v$ and there is an edge between u and v in K). Later in the supplementary information, we will state and prove Lemma 6.1, which gives an algorithm for recovering the unlabeled DAG of K from the structure $S(K)$, implying that the former is *uniquely determined* by the latter. This will complete the proof.

To show that $\Pr[G = K]$ depends only on the unlabeled DAG of K, we derive an explicit expression as follows: for a given vertex v of K, denote the set of neighbors of v that chose v by $N_K(v)$. We call this set the *parents* of v. Furthermore, let $d_1(v) \leq \cdots \leq d_{N_K(v)}(v)$ denote the number of edges from each of the parents of v to v (note that all of this information is given by the unlabeled DAG of K). Then we can write an expression for $\Pr[G = K]$ via the following considerations: this probability is a ratio, the denominator of which is the product $\prod_{j=1}^{mn-1}(2mj)$. This comes from the fact that for the jth connection choice made in the graph, the probability of connecting to *any* vertex contributes a denominator of $2mj$. To determine an expression for the numerator of $\Pr[G = K]$, we consider the contribution of vertex v, for arbitrary v with degree, say, $d > m$. When v joined the graph, it was given degree m. When a parent of v chooses to connect to v, bringing its degree from some d' to $d' + 1$, this contributes a factor of d', leading to a factor of $m(m+1)\cdots d = \frac{d!}{(m-1)!}$. However, more is needed: a given parent that chooses v, say, $d_i(v)$ times can do so in $\binom{m}{d_i(v)}$ ways. More precisely, if a given vertex chooses to connect to ℓ vertices $a_1, \cdots, a_\ell$ times, respectively, where $a_1 + \cdots + a_\ell = m$, then there are $\binom{m}{a_1, \cdots, a_\ell}$ ways to do this. In the expression for the numerator of $\Pr[G = K]$, we will factor out the $m!$ and associate the factor

$\frac{1}{a_i!}$ with its corresponding vertex (so $\frac{1}{d_i!}$ becomes associated with the vertex v). All of this leads to the following expression:

$$\Pr[G = K] = \prod_{j=1}^{mn-1} (2mj)^{-1} \times (m!)^{n-1} \times$$

$$\prod_{d>m} \prod_{v\,:\,\deg_K(v)=d} \frac{d!}{(m-1)!} \prod_{i=1}^{N_K(v)} \frac{1}{d_i!}$$

Here, $\deg_K(v)$ denotes the degree of vertex v in the graph K. The product indexed by d is over all degrees $> m$ present in the graph K, the product indexed by v is over all vertices having the given degree, and the one indexed by i is over all parents of v. Since all parts of this expression are dependent only on the structure of $\mathrm{DAG}(K)$, the lemma is proven. □

In particular for preferential attachment graphs, we have the following inapproximability result.

Theorem 3.3. *Let $\mathcal{G}_n$ denote $\mathcal{PA}(n,m)$, with $m \geq 3$. Then we have $R_*(\mathcal{G}_n, d_e) = 1 - o(1)$. Furthermore, for approximate recovery, $R_*(\mathcal{G}_n, d_a) = \Theta(n^2)$. In other words, the recall is at most $1 - \Theta(1)$, and thus no total order estimation algorithm can solve the problem without making a large number of inversion errors.*

Proof. Recall that $\tau(\cdot,\cdot)$ denotes the Kendall τ distance between two permutations.

We will consider preferential attachment graphs, and will prove the bound for approximate recovery, from which all other statements will follow. It is sufficient to exhibit an adversary distribution for which the statement is true. We consider the set of degree-m nodes (denote this set by $D_m(G)$), which, with high probability, has size $|D_m(G)| = \Theta(n)$. Denote by $S_{D_m(G)}$ the set of permutations in S_n which fix all vertices with degree not equal to m. Within this set, there is a subset $R(G)$, with size tending to ∞ with n and such that any two permutations within $R(G)$ have Kendall τ distance at least $\delta = cn^2$.

We consider an adversary that chooses π uniformly at random from $R(G)$.

Consider an arbitrary total order estimator ϕ. The intuition will be as follows: there are many possible graph/permutation pairs $(K, \hat{\pi})$ such that $\hat{\pi}(K) = \pi(G)$, and these $\hat{\pi}^{-1}$ are well-separated. So they must also be well-separated from $\phi(\pi(G))$. This will provide the lower bound on the expected number of errors.

We start by conditioning on the value of $\pi(G)$ and of π. Here, the outer sum is over all graphs K with at least $c'n$ degree-m nodes, for some appropriate fixed $c' > 0$.

$$\mathbb{E}[\tau(\phi(\pi(G)), \pi^{-1})] \geq \sum_K \Pr[\pi(G) = K] \times$$

$$\sum_{\hat{\pi} \in R(K)} \mathbb{E}[\tau(\phi(K), \pi^{-1}) I[\pi = \hat{\pi}] \mid \pi(G) = K].$$

The inner sum becomes

$$\sum_{\hat{\pi} \in R(K)} \tau(\phi(K), \hat{\pi}^{-1}) \Pr[\pi = \hat{\pi} \mid \pi(G) = K]. \tag{3}$$

Now, the probability in this expression is simply $1/|R(K)|$, which is constant with respect to $\hat{\pi}$.

Thus, the inner sum further simplifies to

$$\frac{1}{|R(K)|} \sum_{\hat{\pi} \in R(K)} \tau(\phi(K), \hat{\pi}^{-1}). \tag{4}$$

To lower bound $\tau(\phi(K), \hat{\pi}^{-1})$ in this sum, denote by δ_{min} the quantity

$$\delta_{min} = \min_{\sigma \in R(K)} \tau(\phi(h), \sigma^{-1}), \tag{5}$$

and let σ_{min} denote the optimizing permutation. Now, using the triangle inequality, we have

$$\begin{aligned}\delta \leq \tau(\hat{\pi}^{-1}, \sigma_{min}^{-1}) &\leq \tau(\hat{\pi}^{-1}, \phi(K)) + \tau(\phi(K), \sigma_{min}^{-1}) \\ &= \tau(\hat{\pi}^{-1}, \phi(K)) + \delta_{min}.\end{aligned}$$

We thus have, for all $\hat{\pi} \in R(K)$ not equal to σ_{min},

$$\tau(\hat{\pi}^{-1}, \phi(K)) \geq \max\{\delta - \delta_{min}, \delta_{min}\}. \tag{6}$$

Then we have the following lower bound on (4).

$$\frac{1}{|R(K)|} \sum_{\hat{\pi} \in R(K)} \tau(\phi(K), \hat{\pi}^{-1}) \geq \frac{|R(K)| - 1}{|R(K)|} \max\{\delta - \delta_{min}, \delta_{min}\}.$$

Now, we plug this in as a lower bound on the inner sum of (3), which gives

$$\begin{aligned}&\mathbb{E}[\tau(\phi(\pi(G)), \pi^{-1})] \\ &\qquad \geq \sum_{K} \Pr[\pi(G) = K] \frac{|R(K)| - 1}{|R(K)|} \max\{\delta - \delta_{min}, \delta_{min}\}.\end{aligned}$$

The outer sum is again over the same set of graphs K as in (3).

Now, in order to simplify the final expression, we recall that $|R(K)| \to \infty$ as $n \to \infty$, and $\max\{\delta - \delta_{min}, \delta_{min}\} = \Theta(n^2)$. All of this gives

$$\mathbb{E}[\tau(\phi(\pi(G)), \pi^{-1})] \geq (1 - o(1)) \cdot \Theta(1) \cdot n^2 = \Theta(n^2). \tag{7}$$

This immediately implies the desired upper bound on the precision/recall for the uniform distribution adversary.

We can prove the result for Erdős-Rényi by directly considering the uniform distribution adversary. The details are quite similar, so we omit them.

□

3.1 Maximum likelihood estimation

For the preferential attachment model, a natural way to approach the total order estimation problem is to frame it in terms of maximum likelihood estimation as follows: $\mathcal{C}_{\mathrm{ML}}(H) = \arg\max_{\sigma \in S_n} \Pr[\pi^{-1} = \sigma | \pi(G) = H]$. The following proposition proves that the optimal solution set $\mathcal{C}_{\mathrm{ML}}$ gives a large number of equiprobable solutions and the maximum likelihood formulation is not a useful approach for the problem.

Theorem 3.4. *The maximum likelihood estimation solution set* $\mathcal{C}_{\mathrm{ML}} = \mathcal{C}_{\mathrm{ML}}(\pi(G))$ *satisfies* $|\mathcal{C}_{\mathrm{ML}}| = e^{n\log n - O(n\log\log n)}$ *with high probability.*

Proof. First, by definition of $\Gamma(H)$, we must have that $\mathcal{C}_{ML}(H) \subseteq \Gamma(H)$. We will show, in fact, that the likelihoods given to all elements of $\Gamma(H)$ are equal. This will then imply that $\mathcal{C}_{ML}(H) = \Gamma(H)$. Next, from a result of [11], we have that with high probability $|\Gamma(\pi(G))| = e^{n\log n - O(n\log\log n)}$, which will complete the proof.

So it is sufficient to show that, for each $\sigma \in \Gamma(H)$,

$$\Pr[G = \sigma(H) | \pi(G) = H]$$

depends only on the structure $S(H)$. To do this, note that by definition of $\mathrm{Adm}(H)$ and $\Gamma(H)$, we must have $\sigma(H) \in \mathrm{Adm}(H)$. So it is enough to show that for any two positive-probability isomorphic graphs G_1 and G_2, we have $\Pr[G = G_1] = \Pr[G = G_2]$. This is the content of Lemma 3.1. This completes the proof of the proposition. □

4 Formulation and Solution of an Optimization Problem

Remark 4.1. We could have defined a tradeoff curve between precision and recall, but this would have the undesirable feature that the optimal precision is not necessarily defined for every value of recall in $[0, 1]$, since some values for recall are not achievable [12].

4.1 Reduction of rational integer program to linear program

It is a general result that a rational linear program such as ours may be solved by converting to an equivalent truly linear program. We define a new variable s, which will capture the rational part of the objective function (i.e., we make the substitution $s = 1/\sum_{1\le u\ne v\le n} x_{u,v}$, and $y_{u,v} = s x_{u,v}$). The objective function is rewritten as a linear function of the normalized variables. This gives us the LP relaxation

$$\max_{y,s} \sum_{1\le u<v\le n} p_{u,v}(H) y_{u,v}, \tag{8}$$

subject to the constraints

1. $y_{u,v} \in [0, s], \forall u, v \in [n]$
2. $0 \le s \le \frac{1}{\varepsilon\binom{n}{2}}$
3. $y_{u,v} + y_{v,u} \le s, \forall u, v \in [n]$
4. $y_{u,v} + y_{v,w} - y_{u,w} \le s, \forall u, v, w \in [n]$
5. $\sum_{1\le u<v\le n} y_{u,v} = 1.$

4.2 Coefficients of optimal precision

The integer program in the Main Text is rather explicit, except for the coefficient $p_{u,v}(H)$. The next lemma gives a combinatorial formula for the probability $p_{u,v}(H)$.

Lemma 4.1 (Coefficients of the optimal precision)**.** *For all $v, w \in [n]$ and graphs H,* $\Pr[\pi^{-1}(v) < \pi^{-1}(w)|\pi(G) = H] = \frac{|\{\sigma \,:\, \sigma^{-1} \in \Gamma(H), \sigma^{-1}(v) < \sigma^{-1}(w)\}|}{|\Gamma(H)|}$.

Proof. We can express the conditional probability in question as a sum, as follows:

$$\begin{aligned}
&\Pr[\pi^{-1}(v) < \pi^{-1}(w)|\pi(G) = H] \\
&= \sum_{\substack{\sigma \,:\, \sigma^{-1} \in \Gamma(H) \\ \sigma^{-1}(v) < \sigma^{-1}(w)}} \Pr[\pi = \sigma|\pi(G) = H]
\end{aligned}$$

Now, recall that $\pi^{-1} \in \Gamma(H)$ under this conditioning, since $\pi(G) = H$ and G is admissible. Moreover, it is uniformly distributed on $\mathrm{Iso}(G, H)$ (the set of isomorphisms from G to H), so we have

$$\begin{aligned}
\Pr[\pi = \sigma|\pi(G) = H] &= \frac{\Pr[G = \sigma^{-1}(H)|\pi(G) = H]}{|\mathrm{Aut}(H)|} \\
&= \frac{1}{|\mathrm{Aut}(H)||\mathrm{Adm}(H)|}.
\end{aligned}$$

Taking the sum, this becomes

$$\frac{|\{\sigma \;:\; \sigma^{-1} \in \Gamma(H), \sigma^{-1}(v) < \sigma^{-1}(w)\}|}{|\mathrm{Aut}(H)||\mathrm{Adm}(H)|}.$$

Finally, recall that $|\mathrm{Adm}(H)| = |\Gamma(H)|/|\mathrm{Aut}(H)|$ [12]. □

5 Optimal Solution for Preferential Attachment Graph Model

From now on, we focus on the preferential attachment graph model, and solve the optimization problem explained in the previous section. In particular we describe how estimate $p_{u,v}(H)$. Later in the section, we provide some efficient estimators that provide good approximations for the optimal solution.

We remark that the existence of self-loops on the initial vertex in preferential attachment graphs allows for clean proofs; our theoretical and empirical results extend without significant changes to models in which these self-loops are not present.

5.1 Recovering edge directions

To efficiently calculate $p_{u,v}(H)$, we need to further characterize the set $\Gamma(H)$ (note that its cardinality is invariant under relabeling). At a high level, given $H = \pi(G)$, we can recover a natural directed, acyclic version $\mathrm{Dir}(H)$, which induces a partial order on its vertices. We can then show that $\Gamma(H)$ is precisely the set of *linear extensions* [3] of this partial order. We can then use algorithms [2, 10] developed for approximate counting of linear extensions to estimate $p_{u,v}(H)$.

We first formalize the DAG in question:

Definition 5.1 (DAG of G)**.** For G distributed according to the preferential attachment distribution (for any m), we define $\mathrm{DAG}(G)$ to be the directed acyclic graph defined on the same vertex set as G, such that there is an edge from u to $v < u$ if and only if there is an edge between u and v in G. This is just G with the directions of edges marked in accordance with the graph evolution (leading from younger nodes to older nodes).

We note here that $\mathrm{DAG}(G)$ and $\pi(\mathrm{DAG}(G))$ are exactly the same in structure and relabeling will not affect the directions, and we denote $\pi(\mathrm{DAG}(G))$ by $\mathrm{Dir}(H)$.

5.2 Integer program coefficients via $\mathrm{Dir}(H)$

The discussion in the previous subsection particularly implies that $\Gamma(H)$ is precisely the set of linear extensions of the partial order defined by $\mathrm{Dir}(H)$.

Coming back to computing the coefficients $p_{u,v}(H)$ in the integer program, we see that this task can be reduced to the problem of counting linear extensions of $\mathrm{Dir}(H)$ and of $\mathrm{Dir}(H)$ with the additional relation that $u \prec v$.

In full generality, the problem of counting linear extensions of an arbitrary partial order is classically known to be #P-complete [4]. However, there exist fully polynomial-time approximation schemes for the problem, which allow us to approximate the coefficients up to an arbitrarily small relative error [8, 9].

Proposition 5.1. *There exists a randomized algorithm which, on input H and positive number λ, outputs a sequence $\hat{p}_{u,v}(H)$ satisfying $|\hat{p}_{u,v}(H) - p_{u,v}(H)| \leq \lambda p_{u,v}(H)$ for all $u, v \in [n]$ with probability $1 - o(1)$, in time*

$$O(n^5 (\log n)^3 \lambda^{-2} \log(1/\lambda)).$$

The time complexity given in the above proposition is based on the worst case running times of the fastest known schemes [2, 10].

Given that we can approximate the coefficients $p_{u,v}(H)$ by $\hat{p}_{u,v}(H) = (1 \pm \lambda) p_{u,v}(H)$ uniformly for arbitrarily small $\lambda > 0$, the next lemma bounds the effect of this approximation on the optimal value of the integer program.

Lemma 5.1. *Consider the integer program whose objective function is given by $\hat{J}_{\varepsilon,\lambda}(\phi) = \frac{\sum_{1 \leq u < v \leq n} \hat{p}_{u,v}(H) x_{u,v}}{\sum_{1 \leq u \neq v \leq n} x_{u,v}}$, with the same constraints as in the original IP. Let ϕ_* and $\hat{\phi}_*$ denote optimal points for the original and modified integer programs, respectively. Then $|\hat{J}_{\varepsilon,\lambda}(\hat{\phi}_*) - J_\varepsilon(\phi_*)| \leq 3\lambda$, for arbitrary $\lambda > 0$.*

Proof. Our goal is to upper bound

$$|J_\varepsilon(\phi_*) - \hat{J}_{\varepsilon,\lambda}(\hat{\phi}_*)|.$$

We can rewrite this as

$$\begin{aligned}
&|J_\varepsilon(\phi_*) - \hat{J}_{\varepsilon,\lambda}(\phi_*) + \hat{J}_{\varepsilon,\lambda}(\phi_*) - J_\varepsilon(\hat{\phi}_*) + J_\varepsilon(\hat{\phi}_*) - \hat{J}_{\varepsilon,\lambda}(\hat{\phi}_*)| \\
&\leq |\hat{J}_{\varepsilon,\lambda}(\hat{\phi}_*) - J_\varepsilon(\hat{\phi}_*)| + |\hat{J}_{\varepsilon,\lambda}(\phi_*) - J_\varepsilon(\phi_*)| \\
&+ |\hat{J}_{\varepsilon,\lambda}(\phi_*) - J_\varepsilon(\hat{\phi}_*)|
\end{aligned}$$

Now, the first and second differences on the right-hand side are at most λ, since

$$\begin{aligned}
&|\hat{J}_{\varepsilon,\lambda}(\phi_*) - J_\varepsilon(\phi_*)| \\
&\quad \le \frac{\sum_{1\le u\ne v\le n} \phi_{*u,v} |p_{u,v}(H) - (1\pm\lambda)p_{u,v}(H)|}{\sum_{1\le u\ne v\le n} \phi_{*u,v}} \\
&\quad \le \lambda \frac{\sum_{1\le u\ne v\le n} \phi_{*u,v}}{\sum_{1\le u\ne v\le n} \phi_{*u,v}} = \lambda.
\end{aligned}$$

The remaining difference can be estimated as follows:

$$\begin{aligned}
&|\hat{J}_{\varepsilon,\lambda}(\phi_*) - J_\varepsilon(\hat{\phi}_*)| \\
&\quad \le \max\{|\hat{J}_{\varepsilon,\lambda}(\hat{\phi}_*) - J_\varepsilon(\hat{\phi}_*)|, |\hat{J}_{\varepsilon,\lambda}(\phi_*) - J_\varepsilon(\phi_*)|\} \\
&\quad \le \lambda.
\end{aligned}$$

This inequality is a result of the fact that ϕ_* and $\hat{\phi}_*$ are optimal points for their respective objective functions. So we took the larger of $\hat{J}_{\varepsilon,\lambda}(\phi_*)$ and $J_\varepsilon(\hat{\phi}_*)$ and increased it, leading to the upper bound.

This shows that

$$|J_\varepsilon(\phi_*) - \hat{J}_{\varepsilon,\lambda}(\hat{\phi}_*)| \le 3\lambda,$$

so we only incur a small additive error in the optimal precision by estimating the coefficients. □

Given these results, we can efficiently upper bound the optimal precision for any given density constraint as follows: on a randomly generated input graph H, we recover its edge directions (as explained in the next section) and use them to approximate the coefficients $p_{u,v}(H)$ up to some relative error λ. Now, at this point, we have a rational linear integer program with our approximation for $pu, v(H)$. We can convert this to an equivalent truly linear integer program using a standard renormalization transformation and consider the natural linear programming relaxation, obtained by replacing the binary constraint with $x_{u,v}(H) \in [0, 1]$ for all u, v. This can be solved in polynomial time using standard tools.

6 Algorithms and Estimators

6.1 Exact recovery of $\pi(\mathrm{DAG}(G))$

Next, we show that we can efficiently recover $\pi(\mathrm{DAG}(G))$ for a preferential attachment graph G, given access to $H = \pi(G)$, via a method that we call the Peeling technique. Recovering the directions of edges from a younger node to an older node is the first step toward our goal. The algorithm starts by identifying the lowest-degree nodes (in our model, the nodes of degree exactly m) and we group them in a bin (group). Then, it removes all of these nodes and their edges from the graph. Importantly, note that, in this step, *all* of the lowest-degree nodes are identified before any of them are removed, and all of the identified nodes are removed together. The process proceeds recursively, removing the lowest-degree nodes and placing them in a new bin, until there are no more nodes to remove from the graph, as summarized in Algorithm 1 below. To construct $\mathrm{Dir}(H) = \pi(\mathrm{DAG}(G))$ during this process, we simply note that all of the edges of a given degree-m node in a given step of the peeling process must be to older nodes; hence, their orientations can be recovered (see Lemma 6.1).

Algorithm 1 Peeling Technique

procedure Peeling(H)
  $t \leftarrow 1$
  while NoNodes(H) > 0 **do** ▷ Finds no. of nodes
    MinDeg $\leftarrow$ FindMinDeg(H) ▷ Finds minimum degree
    $\mathcal{B}_t \leftarrow \{u : \deg(u) = \text{MinDeg}\}$ ▷ Nodes with minimum degree
    $H \leftarrow$ RemoveNodes($H, \mathcal{B}_t$) ▷ Removes minimum degree nodes from H
    $t \leftarrow t + 1$
  end while
  ReverseOrder($\{\mathcal{B}_t\}$) ▷ Reverse the indices of $\mathcal{B}_t$'s, e.g, 1 to t, t to 1
  return $\{\mathcal{B}_t\}$ ▷ Returns set of bins
end procedure

Lemma 6.1. *The* Peeling *algorithm exactly recovers* $\pi(\text{DAG}(G))$ *from* $\pi(G)$.

Proof. The Peeling algorithm maintains the following invariant at the beginning of each step: every degree-m node connects only to vertices older than itself in the remaining graph. This is clear in the initial step, since a node can only have degree exactly m in the full graph if its neighbors are all older than it is. In subsequent steps (assuming by induction that the invariant holds for all previous ones), if some edge incident with a degree-m node u is incident with a younger node v in the current graph, then this implies that some node w older than u and adjacent to u in a previous step has already been removed. This, in turn, implies that in that previous step, w had degree m and was connected to u, a younger vertex. This yields a contradiction, completing the proof. □

The directed, acyclic graph $\pi(\text{DAG}(G))$ conveniently encodes the set of all vertex pairs whose true order relationship can be inferred with *complete certainty*. To make this precise, for a graph H or the resulting DAG $\text{Dir}(H)$, we define a *vertex pair order event* (u, v), for vertices $u, v \in H$, to be simply an ordered pair of distinct vertices. We interpret this as a claim that u came before v according to π^{-1}.

Definition 6.1. A vertex pair order event (u, v) is *perfect* for a graph H if, for all $\sigma \in \Gamma(H)$, we have $\sigma(u) < \sigma(v)$. Equivalently, for any random permutation σ, $\Pr[\sigma^{-1}(u) < \sigma^{-1}(v) | \sigma(G) = H] = 1$.

The following result formalizes our intuition that $\text{Dir}(H)$ captures all probability-1 information about vertex ordering in H:

Theorem 6.1 ($\text{Dir}(H)$ captures perfect vertex pair information)**.** *Consider a graph H on the vertex set $[n]$ satisfying $\Gamma(H) \neq \emptyset$. Let its DAG $\text{Dir}(H)$ be denoted by K. For any $u, v \in [n]$, the pair (u, v) is perfect for H if and only if there exists a directed path in K from v to u (denoted by $v \rightsquigarrow u$).*

Proof. First, we will show that if $v \rightsquigarrow u$, then (u, v) is perfect. This follows by showing the simpler claim that if there is an edge from v to u (denoted by $v \to u$), then (u, v) is perfect.

Let $\sigma \in \Gamma(K)$. This means that $\sigma(K)$ is a positive-probability DAG under the preferential attachment model. Note also that $\sigma(v) \to \sigma(u)$ in $\sigma(K)$, which implies that we certainly must have $\sigma(v) > \sigma(u)$ (vertices only choose to connect to older vertices). Since σ was arbitrary, we have that (u, v) is perfect for H, as desired.

Now we show the converse claim: if (u, v) is perfect for K, then $v \rightsquigarrow u$ in K. We will do this by proving the contrapositive: assume that there is no directed path from v to u. Then we will

construct a permutation σ satisfying i) $\sigma \in \Gamma(K)$, and ii) $\sigma(u) > \sigma(v)$. This is equivalent to producing a feasible *schedule* of the vertices of K (i.e., a sequence of distinct vertices $v_1, v_2, ..., v_n$ of K, such that, for each $j \in [n]$, all m of the the descendants of v_j in K are contained in the set $\{v_1, ..., v_{j-1}\}$). We will require that $v_u > v_v$ in the schedule. Such a schedule gives a permutation satisfying the properties above as follows: for each j, $\sigma(j) = v_j$.

To do this, we start by considering the sub-DAG K_v, consisting of v and all of its descendants. Now, we set v_1 to be the bottom vertex of K (which is also the bottom vertex of K_v). We will add subsequent vertices to our schedule as follows: at each time step $t > 1$, $[n]$ is partitioned into three parts: $S_{p,t}$ (the vertices already in the schedule), $S_{a,t}$ (the *active* vertices; i.e., those vertices not in $S_{p,t}$, all of whose children in K are contained in $S_{p,t}$), and $S_{d,t}$ (the *dormant* vertices; i.e., those vertices that are not active or already processed). So $S_{p,1} = \{v_1\}$, $S_{a,1}$ is the set of neighbors of v_1, and $S_{d,1}$ consists of the rest of the vertices.

We observe that $S_{a,t}$ is nonempty unless $t = n$: otherwise, there are less than n vertices in $S_{p,t}$ (in fact, precisely t of them), and the rest are in $S_{d,t}$. In this case, consider the *lowest* vertex ℓ in $S_{d,t}$; ℓ cannot have any children in $S_{d,t}$, since it is the lowest, so all of its children must be in $S_{p,t}$. But this means precisely that $\ell \in S_{a,t}$. Thus, $S_{a,t}$ must be nonempty.

Note that it is clear that at any time t, we can designate any active vertex as the next one in our schedule; we would then move it to the processed set, potentially resulting in some vertices in $S_{d,t}$ becoming active.

Now, observe that at time $t = 1$, some vertex from K_v must be active (by the same reasoning that established that the active set must be nonempty). In fact, until all vertices of K_v have been processed, there remains at least one such vertex that is active. We thus choose active vertices K_v until it is entirely processed (note that we do not process the vertex $u \notin K_v$ yet, since there is no directed path from v to u). Then we process active vertices until a complete schedule has been generated. By construction, v comes earlier in the schedule than u, which completes the proof. □

6.2 Estimators and their properties

Due to the high polynomial time complexity involved in solving the optimal scheme (Proposition 5.1 and complexity of linear programming), we now provide efficient estimators whose performance is close to the optimal curve. In fact, the linear program itself does not yield an optimal scheme (one has to do a rounding step, which only yields an approximation) or an estimator, but only an upper bound on the optimal precision. Moreover, converting it to an optimal estimator is computationally difficult and thus proposing efficient heuristics is warranted.

The time complexity of the estimators is dominated by the DAG construction, and is $O(n \log n)$.

Note that one must take care in designing an estimator to ensure that the resulting set of vertex pair order relations satisfies transitivity and antisymmetry.

6.2.1 Maximum-density precision 1 estimator

The estimator itself takes as input a graph H and outputs the partial order as $\mathrm{Dir}(H)$ as recovered by the PEELING algorithm. This estimator gives the maximum density among all estimators that have precision one.

In this subsection, we show that the point (density, precision) $= (0, 1)$ is a point on the optimal precision curve: namely, we prove that the perfect-precision estimator has precision one, but asymptotically negligible density, and this estimator has the maximum density among all precision

one estimators. From Theorem 6.1, we know that this achieves precision 1. So, in order to prove the remainder of our claim, we need to analyze the density of this estimator; that is, we analyze the typical number of perfect pairs, culminating in the following theorem.

Theorem 6.2 (Typical number of perfect pairs)**.** *With high probability, for arbitrary fixed $m \geq 1$, the number of perfect pairs associated with G is $\Omega(n \log n)$ and $o(n^2)$ (uniformly in m). When $m = 1$, we have the matching upper bound of $O(n \log n)$, where the hidden constant in the asymptotic notation can be explicitly calculated.*

Proof. **Upper bound:** Let X_t denote the number of perfect pairs in the graph immediately after time t. We will prove the claimed upper bound by upper bounding X_t in expectation, then using Markov's inequality. To bound $\mathbb{E}[X_t]$, we will show that it is sufficient to upper bound $X(v)$ in expectation for each $v < t$, where $X(v)$ denotes the number of descendants of v in the DAG.

Using Proposition 1 of [11] with $\ell = \Theta(\log^{4/5} n \log(\log n))$, we can show that the total number of descendants $X(u)$, for all $u \leq n$, is at most $O(n/\log^{1/5} n)$, with high probability.

Now, we translate this to an upper bound on X_t as follows: we have $\mathbb{E}[X_t] \leq \mathbb{E}[X_{t-1}] + m + mO(t/\log^{1/5} t)$. This upper bound is from the following facts: at time t, all perfect vertex pairs from time $t-1$ are still perfect, contributing the X_{t-1} term. Next, vertex t makes at most m choices, creating at most m new perfect pairs (which explains the second term). Finally, the third term comes from the fact that if t chooses v, and u is a descendant of v (so that (u, v) is a perfect pair), then (u, t) is also perfect.

Solving this recurrence, we find that $\mathbb{E}[X_t] = o(t^2)$, as desired, and the proof is completed using Markov's inequality.

In the case where $m = 1$, we have a much better upper bound on $X(v)$, for arbitrary v: with high probability, at time t, $X(v) = O(\log t)$, as a result of the height of the tree being $O(\log t)$. This gives the improved bound of $\mathbb{E}[X_t] = O(t \log t)$.

Lower bound: To prove the lower bound of $\Omega(t \log t)$ perfect pairs, we write the total number of perfect pairs in such a graph as the sum, over all vertices $v \leq t$, of the number of descendants $X(v)$: $X_t = \sum_{v \leq t} X(v)$.

In the $m = 1$ case (where G is a tree), this implies that X_t is the *total path length* of the tree. It is known that, with high probability, this parameter is $\Theta(t \log t)$ (since it can be written as an additive parameter, the results of [16] apply).

Now, to prove it for general m, we recall that to form a sample G from $\mathcal{PA}(n, m)$, we first produce a sample T from $\mathcal{PA}(mn, 1)$, then collapse sequences of m consecutive vertices. The number of perfect pairs in G is at least the same quantity in T, divided by m^2, since there can be at most m^2 edges from one sequence of m consecutive vertices to an older sequence in T. Using the result for $m = 1$, we have that with high probability, the number of perfect pairs in T is $\Theta(mt \log(mt)) = \Theta(t \log t)$, which implies that the number of perfect pairs in G is $\Omega(t \log t)$. □

The above theorem implies that the density of the perfect pair estimator is asymptotically 0, for arbitrary m. This is validated by simulation as shown in Figure 1. [1]

Further scrutiny of the numerical evidence leads to a conjecture about the more precise behavior of the number of perfect pairs as a function of m: we conjecture that for $m > 1$, the number of perfect pairs is $O(n^{1+\delta(m)})$, for some function $1 > \delta(m) > 0$ (see again Figure 1).

[1] All simulation results take empirical expected values with respect to several samples from the graph distribution in question.

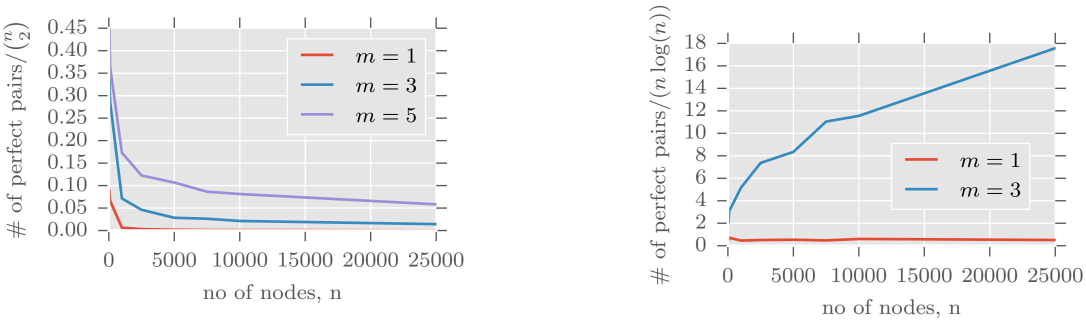

(a) Upper bound

(b) Lower bound

Figure 1: Normalizations of the number of perfect pairs in synthetic preferential attachment graphs.

This conjecture arises from the intuition from the (rigorous) proof of Theorem 6.2 as follows: denote by $X(u)$ the number of descendants of any vertex u in DAG(G), and by X_t the number of perfect pairs in the graph up to time t. If we can upper bound the number of descendants of any given vertex at time t by $O(t^c)$, for some constant $c = c(m) < 1$ dependent only on m (which is supported by empirical evidence), then we have $\mathbb{E}[X_t] \leq \mathbb{E}[X_{t-1}] + m + O(t^c) = \mathbb{E}[X_{t-1}] + O(t^c)$. This upper bound arises as follows: the pairs that are perfect at time $t - 1$ remain perfect at time t, yielding the first term of the upper bound. The second and third terms arise from the fact that vertex t connects to at most m other vertices (resulting in m additional perfect pairs), and each perfect pair (v, w) involving two descendants of a node chosen by vertex t yields another perfect pair (v, t).

Iterating this recurrence and using Euler-Maclaurin summation, we find that $\mathbb{E}[X_t] = O(t^{1+c(m)})$, as desired. We give empirical evidence for the upper bound on $X(t)$ (and hence for the improved upper bound on the number of perfect pairs) in Figure 2, which indicates that the maximum number of descendants of any node at a given time t is $O(t^{c(m)})$.

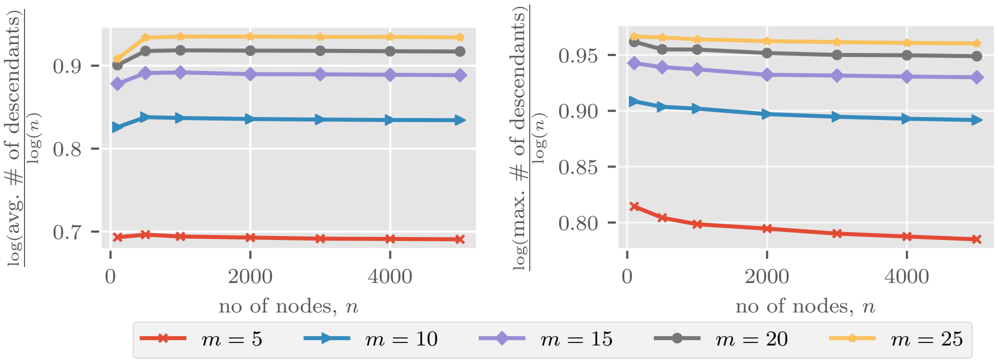

Figure 2: Normalized average and maximum number of descendants of degree-m nodes in DAG(G).

Theorem 6.2 gives us a single point on the optimal precision curve, and it is very simple to show that the curve is decreasing as ε increases. In the next section, we show Peeling estimator achieves nontrivial density and precision with high probability, which gives a lower bound on the optimal precision for another value of the density. We will use this to give an empirical indication of the

tightness of our upper bound.

6.2.2 Peeling: A linear binning estimator via peeling

When the term Peeling is used as an estimator, we mean the linear binning estimator[2] from the bins (groups of nodes) given by the Peeling Algorithm 1. In particular, the sequence of subsets of vertices removed during each step naturally gives a partial order: each such subset forms a bin, and bins that are removed earlier are considered to contain younger vertices.

We can start by showing a crude estimate of the precision and density of the Peeling estimator.

Theorem 6.3 (Non-negligible precision and density of the peeling estimator)**.** *For each* $m \geq 1$ *the* Peeling *estimator has precision and density* $\Theta(1)$.

Proof. The precision claim follows by showing that, with high probability, there exist $\Theta(n)$ vertices in the last $\Theta(n)$ timesteps that are never chosen (so that they are removed in the first bin and, thus, correctly declared to be younger than $\Theta(n)$ other vertices). This also proves the density claim.

The required result follows easily from the fact that with high probability there are $\Theta(n)$ vertices of degree m in the graph of size n. Namely, suppose that there are J degree-m vertices. We consider the $J/2$th such vertex (say, vertex v). Then, clearly, since $J/2 = \Theta(n)$, we have that $n - J/2$ is also $\Theta(n)$, so that the result follows. □

We now discuss further analysis of the procedure. In the case $m = 1$, the original graph G is a tree, and we are able to precisely characterize certain parameters of the Peeling procedure, owing to the large amount of literature surrounding the preferential attachment distribution for this case (the structure is (essentially) identically distributed to a random *plane-oriented recursive tree*): for instance, the asymptotic size of the jth youngest bin, for each fixed j, may be precisely determined by writing it as an additive parameter and using results from Wagner et al. [16]. This gives us a complicated but explicitly computable convergent series expression for the typical asymptotic density of the estimator in this case. Precise theoretical results for $m > 1$ (and for the precision in the $m = 1$ case) are much more elusive, owing to the fact that the precision and the DAG structure of the graph fail to lead to clean recursive formulas for parameters of interest (though a Pólya urn approach allows us to derive very complicated expressions involving arbitrarily high moments and covariances of quantities of interest); however, we can show that the peeling process recovers all perfect pairs, in addition to many imperfect ones.

7 Experiments

In this section, we evaluate our methods on synthetic and real-world networks. In what follows, $\sigma_{\text{perf}}, \sigma_{\text{peel}}, \sigma_{\text{peel+}}$ denote the partial orders produced by the Perfect-precision, Peeling, and Peeling+ algorithms.

7.1 Synthetic graphs

We derive significant insight by studying the Peeling method empirically. Table 1 and Figure 3

[2] A *Linear binning* is a special type of partial order, defined as follows: a partial order $\prec$ is a linear binning if its elements may be partitioned into subsets $\mathcal{B}_1, ..., \mathcal{B}_j$, for some j, such that $u \prec v$ if and only if there is a pair $i < j$ such that $u \in \mathcal{B}_i$ and $v \in \mathcal{B}_j$. This is equivalent to a graded partial order.

m	$\theta(\sigma_{\text{peel}})$	$\rho(\sigma_{\text{peel}})$	$\delta(\sigma_{\text{peel}})$
5	0.954	0.758	0.794
10	0.971	0.858	0.884
25	0.986	0.936	0.949
50	0.993	0.967	0.974

Table 1: Comparison for $\mathcal{PA}(n = 5000, m)$

shows simulation results for samples from the Barabási-Albert model. The performance improves greatly with even small increases in m. This can be explained intuitively as follows: for small m, as the graph evolves, each new vertex is likely to connect to high-degree nodes, which are already in older bins in DAG(G). Thus, bins tend to be large, resulting in low precision and recall. For larger m, each new node chooses a degree-m node (say, v) with higher probability, since a constant fraction of all nodes have degree m and the number of choices that each new vertex makes increases with m. A new vertex choosing v results in shifting the bin indices of all descendants of v. Thus, larger m leads to a DAG with a more equitable distribution of vertices across bins, which yields higher precision and recall.

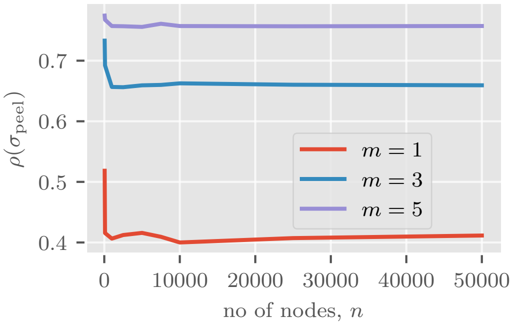

Figure 3: Recall of the PEELING algorithm remains constant when the number of nodes varies

7.2 Real-world networks

All datasets used in our study, except the human brain networks, are publicly available. We explain later how the brain network is formed. The ArXiv, Wikipedia and DBLP are collected from [5]. The first three networks have partial temporal information available, and the original ranking of the network is deduced from the time stamps of the edges. For the brain networks, no explicit temporal information is available.

The ArXiv network. Directed network; the nodes are publications and an edge indicates one publication citing another. The original ranking is not a full ranking; it has 1457 bins.

The Simple English Wikipedia dynamic network. Directed network; it shows the evolution of hyperlinks between articles of the Simple English Wikipedia. Nodes represent articles and an edge indicates that a hyperlink was added or removed.

DBLP computer science bibliography:. Undirected network; an edge between two authors represents a common publication.

Human brain network - Cambridge-Buckner. It is an undirected network. The nodes here

are the regions in the human brain, and the edges represent communication between two regions while performing an activity. The network is formed as follows. We collect the human brain fMRI data at resting state, from the Cambridge-Buckner dataset shared by NITRC[3]. An initial network is first formed with nodes as voxels, and there are $243,648$ voxels. Each voxel is associated with a time series data that lasted approximately 350 seconds. We compute the Pearson correlation coefficient between the time series of every pair of voxels. If the correlation is greater than 0.8 we form an edge between the voxels. Finally we form a network of 56 regions, which is a contracted network of voxels - all voxels corresponding to a region are merged to form a node, and an edge is created between two regions when there exists at least one edge between member voxels.

Human brain network - Human Connectome project. This is the resting state fMRI data from Human Connectome project focusing on the cortex area. The Human Connectome project provides a clean and refined data, which gives consistent results in many published studies. We processed and formed brain networks out of 100 healthy young adults. First, the cortex brain data corresponding to 100 subjects (2 sessions per subject, and 2 scans per session) is parcellated into 180 regions per hemisphere using a procedure described in [7]. Then the correlation matrices are formed from the time series of the parcellated data. Finally binary, undirected networks are constructed from the correlation matrices as follows: a spanning tree is created first from the complete network of the correlation matrix, and later k-nearest neighbors (higher correlation values) of each node are added into this network, where k is chosen as 10 in our case (see [1] for different ways to generate networks from the brain data).

Each network has 300 nodes, which are regions or clusters formed from group-Independent Component Analysis. The data is in correlation matrix format, with each element as the Gaussianized version of the Pearson correlation coefficient (Fisher Z transform with AR(1) process estimation). In order to form a binary adjacency matrix, we use a threshold just high enough to make the resulting graph connected. Such a graph is sparse.

Facebook Wall network. Recently, strategic information spreading in online social networks like Twitter or Facebook is alleged to create biases in the opinions of people, and skews the political elections results. In most cases it is difficult to track the culprits behind the false information. Our methods to rank the nodes according to their arrival finds applications here. In general, in an online social network the identification of malicious information sources and carriers, be it in the case of an online spam spreading in the Internet or a misinformation or rumor propagating, allows timely quarantine of the epidemic-like spreading to mitigate the damage caused. In particular, we study the network of Facebook wall posts here, where nodes are the users and the edges are wall posts between users on the social network Facebook located in the New Orleans region [18]. Any friend of a given user can see all posts on that user's wall, so communication is public among friends. An edge (u, v, t) means that user u posted on user v's wall at time t. We could limit to the network of wall posts containing a specific hashtag, and thus focus on rumor spreading on a certain topic.

SMS-A. Short messaging service (SMS) is a texting service provided on mobile phones. In this dataset, an edge (u, v, t) means that person u sent an SMS message to person v at time t [19].

[3] https://www.nitrc.org

7.2.1 Results

For all real networks above for which an approximate ground truth is known, our algorithms yield excellent precision and recall results, consistent with our theoretical predictions (see Table 2, and Figures 4 and 5) in the main paper). Note that the precision and recall measures achieved on these datasets using our estimators are somewhat pessimistic, due to the fact that the only ground truths available are sparse partial orders. The table shows the performance of the PEELING+ algorithm, in addition to that of PEELING. When the density of the recovered partial order by PEELING algorithm is low, the recall can be improved via PEELING+ with a slight loss in precision (see the Wikipedia result).

For the brain networks, no ground truth is known, so we cannot give measures of precision or recall for the generated orderings. We propose our orderings for further study. We discuss the results on these networks below.

Human brain network - Cambridge-Buckner. The purpose here is to predict the evolutionary order among the important regions inside the brain. Figure 3A (left) presents the bins of regions we found via PEELING algorithm. Figure 3B (right) shows an image of the human brain with prominent regions annotated with bin number. Regarding plausibility of our proposed order, we first note that prior studies support preferential attachment involvement in brain networks [17]. Moreover, the placement of certain brain regions in the ordering are consistent with known results. E.g., the corpus callosum, which joins the two hemispheres of the brain, and the cerebellum and most of its related structures, which are present in all vertebrates, are supposed to have developed at the earliest stages of brain evolution. Another example is the uncinate fasciculus, the last white matter tract to be evolved in the human brain, which is in compliance with our result. We consider this as a first step towards finding a proper evolutionary order of brain regions, and corroborating evidence (which is currently lacking) must come from alternative methodologies devised by domain experts. This analysis could separate regions with core functionality from those involved in functional specializations, giving information about evolution of various structural and functional elements.

Human brain network - Human Connectome project. We analyzed this dataset in order to demonstrate that our methods are robust across networks coming from different individuals. We plot the histogram of arrival rank of prominent auditory, visual, and somatosensory regions. The histograms show a concentration, indicating consistency in ranking of the respective regions. Moreover, we observe that most of the regions that serve as prime functionaries among auditory, visual and somatosensory regions have consistently early arrival order in the 100 brain networks we took into account. This is in accordance with the typical notion of early arrivals of these regions in the human brain evolution.

References

[1] A. F. Alexander-Bloch, N. Gogtay, D. Meunier, R. Birn, L. Clasen, F. Lalonde, R. Lenroot, J. Giedd, and E. T. Bullmore. Disrupted modularity and local connectivity of brain functional networks in childhood-onset schizophrenia. *Frontiers in systems neuroscience*, 4:147, 2010. 17

[2] J. Banks, S. Garrabrant, M. L. Huber, and A. Perizzolo. Using TPA to count linear extensions. *arXiv preprint arXiv:1010.4981*, 2010. 8, 9

[3] G. Brightwell and P. Winkler. Counting linear extensions. *Order*, 8(3):225–242, 1991. 8

[4] G. Brightwell and P. Winkler. Counting linear extensions is #p-complete. In *Proceedings of the Twenty-third Annual ACM Symposium on Theory of Computing (STOC)*, pages 175–181, New York, NY, USA, 1991. ACM. 9

[5] K. T. K. N. Collection. Preferential attachment exponent. Available on `http://konect.uni-koblenz.de/statistics/prefatt` [Accessed: 30 April 2017], 2016. 16

[6] A. Frieze, W. Pegden, et al. Looking for vertex number one. *The Annals of Applied Probability*, 27(1):582–630, 2017. 2

[7] M. F. Glasser, T. S. Coalson, E. C. Robinson, C. D. Hacker, J. Harwell, E. Yacoub, K. Ugurbil, J. Andersson, C. F. Beckmann, M. Jenkinson, et al. A multi-modal parcellation of human cerebral cortex. *Nature*, 536(7615):171–178, 2016. 17

[8] M. Huber. Fast perfect sampling from linear extensions. *Discrete Mathematics*, 306(4):420–428, 2006. 9

[9] M. R. Jerrum, L. G. Valiant, and V. V. Vazirani. Random generation of combinatorial structures from a uniform distribution. *Theoretical Computer Science*, 43:169–188, 1986. 9

[10] A. Karzanov and L. Khachiyan. On the conductance of order markov chains. *Order*, 8(1):7–15, 1991. 8, 9

[11] T. Łuczak, A. Magner, and W. Szpankowski. Asymmetry and structural information in preferential attachment graphs. *Random Structures & Algorithms (to appear); arXiv preprint arXiv:1607.04102*, 2019. 2, 7, 13

[12] A. Magner, A. Grama, J. Sreedharan, and W. Szpankowski. Recovery of vertex orderings in dynamic graphs. In *Proceedings of the IEEE International Symposium on Information Theory (ISIT)*, 2017. 7, 8

[13] A. Magner, A. Grama, J. K. Sreedharan, and W. Szpankowski. Recovery of vertex orderings in dynamic graphs. In *2017 IEEE International Symposium on Information Theory (ISIT)*, pages 1563–1567, June 2017. 2

[14] A. Magner, J. K. Sreedharan, A. Y. Grama, and W. Szpankowski. Times: Temporal information maximally extracted from structures. In *Proceedings of the 2018 World Wide Web Conference*, WWW '18, pages 389–398, 2018. 2

[15] S. M. V. Mahantesh, S. Iyengar, M. Vijesh, S. R. Nayak, and N. Shenoy. Prediction of arrival of nodes in a scale free network. In *Proceedings of the 2012 International Conference on Advances in Social Networks Analysis and Mining (ASONAM)*, pages 517–521, 2012. 2

[16] D. Ralaivaosaona and S. Wagner. Additive functionals of d-ary increasing trees. In *Proceedings of the 27th International Conference on Probabilistic, Combinatorial, and Asymptotic Methods for the Analysis of Algorithms*, pages 1–12, 2016. 13, 15

[17] P. E. Vértes, A. F. Alexander-Bloch, N. Gogtay, J. N. Giedd, J. L. Rapoport, and E. T. Bullmore. Simple models of human brain functional networks. *Proceedings of the National Academy of Sciences*, 109(15):5868–5873, 2012. 18

[18] B. Viswanath, A. Mislove, M. Cha, and K. P. Gummadi. On the evolution of user interaction in facebook. In *Proceedings of the 2nd ACM workshop on Online social networks*, pages 37–42. ACM, 2009. 17

[19] Y. Wu, C. Zhou, J. Xiao, J. Kurths, and H. J. Schellnhuber. Evidence for a bimodal distribution in human communication. *Proceedings of the national academy of sciences*, 107(44):18803–18808, 2010. 17
